# A systematic review and meta-analysis on the efficacy of intravesical therapy for bladder pain syndrome/interstitial cystitis

**DOI:** 10.1007/s00192-015-2890-7

**Published:** 2015-11-20

**Authors:** Jayanta M. Barua, Ignacio Arance, Javier C. Angulo, Claus R. Riedl

**Affiliations:** King George Hospital (BHRUT), Ilford, UK; Barts and the London School of Medicine & Dentistry, QMUL, London, UK; Servicio de Urología, Hospital Universitario de Getafe, Universidad Europea de Madrid, Madrid, Spain; Department of Urology, Landesklinikum Thermenregion, Wimmergasse 19, 2500 Baden, Austria

**Keywords:** Bladder pain syndrome, Interstitial cystitis, Intravesical instillation, Hyaluronic acid

## Abstract

Bladder pain syndrome/interstitial cystitis (BPS/IC) is a chronic disease characterised by persistent irritating micturition symptoms and pain. The objective was to compare the clinical efficacy of currently available products for intravesical therapy of BPS/IC and to assess their pharmacoeconomic impact. A Pubmed/Medline database search was performed for articles on intravesical therapy for BPS/IC. A total of 345 publications were identified, from which 326 were excluded. Statistical evaluation was performed with effect size (ES) assessment of symptom reduction and response rates. The final set of 19 articles on intravesical BPS/IC therapy included 5 prospective controlled trials (CTs), the remaining were classified as uncontrolled clinical studies. The total number of patients included was 801, 228 of whom had been evaluated in a CT. For CTs, the largest ES for symptom reduction as well as response rate was observed for high molecular weight hyaluronic acid (HMW-HA), with similar findings in two uncontrolled studies with HMW-HA. The number needed to treat to achieve a response to intravesical therapy was 2.67 for intravesical pentosan polysulphate and 1.31 for HMW-HA which were superior to all other instillates. HMW-HA was significantly superior in cost effectiveness and cost efficacy to all other instillation regimes. The present meta-analysis combined medical and pharmacoeconomic aspects and demonstrated an advantage of HMW-HA over other instillation agents; however, direct comparisons between the different products have not been performed to date in properly designed controlled studies.

## Introduction

Bladder pain syndrome/interstitial cystitis (BPS/IC) is a chronic disease characterised by persistent irritating micturition symptoms and pain [1]. While there is no general agreement on the precise pathophysiology of this disease, a disorder at the level of the urine–tissue barrier of the bladder seems to be the underlying mechanism behind the functional, anatomical and symptomatic manifestations in a considerable number of cases.

Even if study results are not entirely consistent and, therefore, the subject is not closed, a defect in the protective bladder’s mucous lining of glycosaminoglycans (GAG) and, thus, the urine–tissue barrier, has been documented in a subset of BPS/IC patients [2], mainly demonstrated by a positive potassium sensitivity test (PST) [3–6] and the favourable response to GAG-restoring agents.

Glycosaminoglycans are classified in four structural families [7] (heparin and heparan sulphates; chondroitin and dermatan sulphates; hyaluronan; and keratan sulphate) and have been used during the last few decades as intravesical instillations for GAG substitution therapy with the benefit of delivering high concentrations of the therapeutic agent at the target tissue with a low risk of systemic side effects [8].

The diversity of available therapeutic agents for GAG substitution may make it difficult for physicians to choose the optimal treatment for their patients [9, 10]; therefore, the selection of a particular therapeutic regimen should be based on its capacity for symptom improvement, its impact on the patient’s quality of life, and its costs [10].

Heparin has commonly been used off-label for BPS/IC therapy. Pentosan polysulphate (PPS), a semisynthetic heparin-like GAG of low molecular weight (MW) classically used for oral therapy of BPS/IC, is also available for intravesical instillation. Today, hyaluronan, the salt of hyaluronic acid (HA), and chondroitin sulphate (ChS) are the two most commonly used GAGs for intravesical treatment, alone or in combination.

Other intravesical instillations containing anaesthetic solutions, such as lidocaine and bupivacaine, are also used in combination with sodium bicarbonate to control bladder pain [11], while dimethyl sulfoxide (DMSO), which has a putative effect on the sensory peripheral nerves of the bladder [12], is the only intravesical therapy approved by the FDA.

At present, eight agents for intravesical BPS/IC therapy are commercially available in Europe (Table 1)

**Table 1:** Intravesical agents for bladder pain syndrome/interstitial cystitis (BPS/IC) therapy, their registered trade names and pharmacological composition

Intravesical agent	Registered trade name	Composition
HA	Cystistat®	40 mg HMW-HA (0.08 %) in 50 ml
	Hyacyst®	40/120 mg HA (0.08/0.24 %) in 50 ml (MW unknown)
	Uromac®	100 mg LMW HA (0.2 %) in 50 ml
CS	Gepan instill®	80 mg CS (0.2 %) in 40 ml
	Uracyst®	400 mg CS (2 %) in 20 ml
HA/CS	Ialuril®	800 mg LMW-HA (1.6 %) / 1 g CS (2 %) in 50 ml
PPS^a^	Cyst-u-ron®	300 mg PPS (1 %) in 30 ml
DMSO	Rimso-50®	27 g DMSO (5.4 %) in 50 ml

*HA* hyaluronan, *CS* chondroitin sulphate, *PPS* pentosan polysulphate sodium, *DMSO* dimethyl sulfoxide, *HMW* high molecular weight, *MW* molecular weight, *LMW* low molecular weight

^a^Elmiron® is the oral form of pentosan polysulphate sodium (100 mg)

Despite the widespread clinical use of each of these substances, the research-based evidence regarding therapeutic efficacy is limited and mainly based on uncontrolled trials. Levels of evidence for the use of these agents have been rated 1b for PPS and heparin, and 2b for HA and ChS in their different concentrations [13].

The aim of this meta-analytical review is to directly compare the data on the clinical efficacy of products currently available for intravesical BPS/IC therapy and to assess their pharmacoeconomic impact to assist in therapeutic decision-making.

## Materials and methods

### Literature search

Preferred Reporting Items for Systematic reviews and Meta-Analyses (PRISMA) [14] guidelines were used to perform a comprehensive search for literature on intravesical therapy for BPS/IC and published in the PubMed/Medline database from 1996 to 2014. The Medical Subject Heading (MESH) search terms used were: interstitial cystitis, bladder pain syndrome, intravesical treatment, intravesical chondroitin sulphate, intravesical hyaluronan, intravesical PPS, intravesical DMSO, and intravesical lidocaine.

### Selection criteria

The PubMed/Medline search allowed us to identify publications regarding intravesical treatment for BPS/IC. From these, only studies in English or Spanish reporting clinical results were reviewed. Further analysis included only studies with a single compound or a fixed commercially available combination. Studies were excluded if they:

1. Were performed using intravesical “cocktails”
2. Did not evaluate intravesical treatment for BPS/IC
3. Assessed other related topics, but did not evaluate treatment efficacy (case reports, conference reports etc.)
4. Were defined as review or meta-analysis papers

### Data extraction and statistical analysis

Data were extracted from each publication by two independent reviewers (CLL and AMG) and included: type of study according to the presence of a control arm (controlled or uncontrolled clinical trials), randomisation (randomised or non-randomised controlled trials) and observational studies (prospective or retrospective), type of intravesical solution, total of patients at baseline (intention-to-treat analysis), total patients treated (per protocol analysis), number of patients lost to follow-up, therapy regimen (total number of instillations/frequency of application), instruments/scales used for evaluation of symptoms before and after treatment, and response rates (RR), considered as the percentage of patients with symptom reduction after therapy out of the total study sample [15]. In the absence of an explicitly cited definition for RR, and according previous publications [16, 17], a reduction of ≥2 on a visual analogue scale (VAS) was considered to be a response to treatment; the percentage of responders was inferred by calculating the z value, defined as the proportion of responders within confidence intervals at 95 % (CI 95 %).

The different products were compared with regard to the average reduction of bladder symptoms on the VAS and the overall response rates by calculation of “Cohen’s d” [18], a statistical value for effect size (ES) based on differences between mean values and the average difference in the proportion of patients with a response to treatment and allows the difference between the two groups to be quantified using the standard deviation. While the broadly accepted and cited *p* value informs whether an effect from the investigated measure exists, it does not reveal the size of the effect. For Cohen’s *d*, a low value < 0.5 shows a small ES, d values > 1 are regarded as large ES.

From the CTs, a post-hoc calculation of a composite VAS/RR odds ratio (OR) allowed the comparison of results of different intravesical agents with the placebo/control-treated arms. These values were also used to calculate the number of patients needed to treat (NNT) to obtain a response.

Finally, the pharmacoeconomic assessment was performed by multiplying the unit costs by the number of instillations administered in each CT. Costs per unit and frequency of instillation (one per week) are very similar for HA and ChS products; thus, a cost factor of 1 has been assigned for these two therapies. PPS is less expensive (instillation units cost 40 % of HA/ChS, oral therapy 20 %), but has to be administered two or three times per week and may be accompanied by oral therapy, Thus, a cost factor of 0.4 for intravesical/0.6 for intravesical and oral therapy has been assumed for PPS trials.

## Results

The Medline search led to a total of 345 hits. Initially, all titles and abstracts were reviewed to identify studies not directly reporting on BPS/IC or intravesical treatment for BPS/IC, reviews, and other type of publications not suitable for this analysis.

From the 33 studies selected according to the outlined criteria, 11 (33.33 %) assessed intravesical therapy with HMW-HA 0.08 % (of which one paper also evaluated intravesical heparin), 7 studies presented the results of ChS 0.2 % therapy (21.21 %), and 3 studies evaluated treatment with ChS 2.0 % (9.09 %). Results of the combined formulation of LMW-HA + ChS 2.0 % were presented in 4 publications (12.12 %), 3 papers assessed the results of intravesical treatment with PPS (9.09 %), 2 with lidocaine (6.06 %), and another 3 articles reported results with DMSO (9.09 %). No publications on LMW-HA alone for the treatment of BPS/IC were retrieved from the search.

The main criteria for the exclusion of studies were reports on combination therapies with non-GAG substances, duplicity of results (same sample of patients), and results not comparable in a standardised way because of the different instruments used for outcome evaluation. Thus, 2 of the articles on HMW-HA therapy were excluded from further analysis because they involved combination with alkalised lidocaine [19] or oral PPS [20]. Similarly, 2 publications on DMSO were excluded because of combination therapies (DMSO + hydrocortisone + heparin sulphate [21] and DMSO + triamcinolone [22]). One study with the combination of LMW-HA 1.6 % + ChS 2.0 % [23] was excluded owing to obvious coincidences with another publication [24].

The majority of the selected publications referred to BPS/IC therapy, except for 2 papers on ChS 0.2 %, which evaluated the product in patients with overactive bladder [25, 26] and for the prophylaxis of radiation cystitis [27]; 2 of the excluded articles reported on the efficacy of a combination of heparin and lidocaine as an acute analgesic intervention for severe pain [28, 29].

**Fig. 1:**
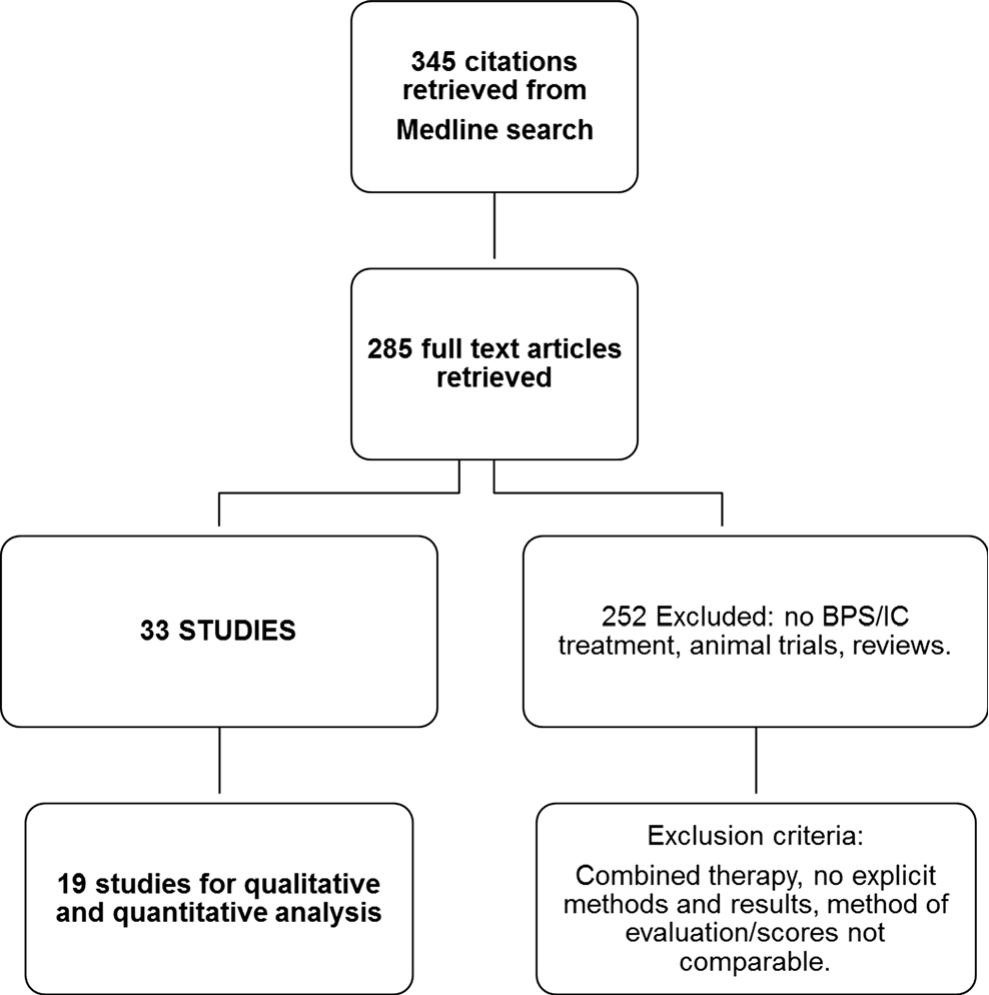
Selection process of the studies included for analysis

Two publications on HMW-HA 0.08 % therapy were excluded because they evaluated symptoms with different non-standardised scales [30, 31], and in one study in which patients received intravesical instillations with LMW-HA 1.6 % + ChS 2.0 % data on VAS pain scores were missing [32]. The full text version of one publication for ChS 0.2 % [33] could not be found after searching several databases. In summary, 14 publications were excluded (Fig. 1).

The final set of 19 articles on intravesical BPS/IC therapy was further qualitatively and quantitatively analysed: 5 studies were prospective controlled trials [34–38], 1 compared different intravesical products [39], 1 paper was designed as a retrospective study [40], and another 1 compared two different regimens of the same product [41]. The rest of the trials were classified as uncontrolled clinical studies (Table 2).

**Table 2:** All studies included for comparison

Study (reference)	Treatment	Type of study	Total sample	Instillation protocol	Follow-up	Evaluation scale
Morales et al. [15]	0.8 % HMW-HA (Cystistat®)	UCT	25	Weekly for 4 weeks and monthly for 12 months	12 months	VAS for pain
Kallestrup et al. [42]		UCT	48	Weekly for 4 weeks and monthly for 2 months	3 years	VAS for pain
Gupta et al. [43]		UCT	38	Weekly for 6 weeks	6 weeks	ICSI-ICSP
Riedl et al. [16]		UCT	121	Weekly and in response to symptoms	6.5 months	VAS for pain
Engelhardt et al. [40]		UCT	70	Weekly for 10 weeks	4.9 years	VAS for pain
Shao et al. [34]		CT	31	After hydrodistention, weekly for 4 weeks and monthly for 2 months	9 months	VAS for pain
Lai et al. protocol A ^a^ [41]		UCT	30	Weekly for 4 weeks and monthly for 5 months	6 months	VAS for pain/ICSI-ICPI
Lai et al. protocol B ^a^ [41]		UCT	30	Every 2 weeks	6 months	VAS for pain/ICSI-ICPI
Steinhoff [44]	0.2 % CS (Gepan instill®)	UCT	18	Weekly for 4 weeks and monthly for 12 months	12 months	ICSI-ICPI
Nordling and van Ophoven [45]		UCT	165	Weekly for 4 to 6 weeks and one monthly	3 months	VAS for pain
Nickel et al. [46]	2 % CS (Uracyst®/Uropol S®)	UCT	53	Weekly for 6 weeks and monthly for 4 months	6 months	VAS for pain/ICSI-ICPI
Nickel et al. [35]		CT	65	Weekly for 6 weeks	3 months	VAS for pain/ICSI-ICPI
Nickel et al. [35]		CT	98	Weekly for 7 weeks	3 months	VAS for pain/ICSI-ICPI
Porru et al. [24]	1.6 % LMW-HA + 2 % CS (Ialuril®)	UCT		Weekly for 12 weeks and biweekly for 6 months	6 months	VAS for pain/ICSI-ICPI
Porru et al. [47]		UCT	22	Weekly for 8 weeks and biweekly for 6 months	6 months	VAS for pain/ICSI-ICPI
Bade et al. [48]	i-PPS 300 mg^b^ (Elmiron®)	UCT	20	Every 2 weeks for 3 months	3 months	Not available
Bade et al. [37]		CT	9	Twice weekly for 3 months	3 months	ICSI/ICPI
Daha et al.[17]		UCT	29	Twice weekly for 10 weeks; monthly for 6 months	12 months	ICSI/ICPI
Davis et al. [38]	i-PPS 200 mg^c^ + daily o-PPS 400 mg (Elmiron®)	CT	41	Twice a week for 18 weeks	18 weeks	VAS for pain/ ICSI-ICPI
Sairanen et al. [39]	DMSO	UCT	37	Weekly for 6 weeks	3 months	VAS for pain

*CT* controlled trials, *UCT* uncontrolled trials, *VAS* visual analogue scale, *ICSI*/*ICPI* O’Leary–Sant symptom index and problem index, *i-PPS* intravesical PPS, *o-PPS* oral PPS

^a^Lai et al. corresponds to two effective protocols with HMW-HA evaluated in the same publication

^b^Intravesical instillation with 300 mg (three capsules) of Elmiron® + mixed with 50 ml of 0.9 % sodium chloride

^c^200 mg or two capsules mixed with 30 ml sterile normal buffered saline

### Patients characteristics

The final sample of 19 trials corresponds to a total of 801 patients, with 228 patients evaluated in a CT. Only 4 trials included male and female patients [15, 35, 44, 45], while the rest of the studies exclusively included women.

Diagnosis of BPS/IC has been made according to NIDDK/National Institutes of Health criteria in most studies (one including cystoscopic examination [42]), except for three publications (ESSIC criteria [40], the East Asian guidelines [41] or clinical perception plus cystoscopy [38]).

Other relevant differences were also detected: six of the studies included “treatment-refractory patients”, considered to be cases with an inadequate response to previous BPS/IC treatments such as intravesical DMSO [15], heparin and/or PPS [15, 41] and/or oral drug therapy [24, 43, 47]. One study only included “naïve patients” (no previous disease-specific treatment) with a positive PST and a reduction of ≥ 2 points in symptom score after the first instillation of ChS 0.2 % [44].

### Instillation regimen

Initial instillation therapy was performed weekly in 73.7 % of the studies, but with different follow-up schedules (Table 2). The instillation procedure was similar in most studies, with the exception of one trial, where a solution of lidocaine and sodium bicarbonate was instilled before the PPS to reduce the procedure-related discomfort and to improve the retention of the subsequently instilled PPS [38].

### Evaluation of efficacy

In most studies a first symptom evaluation was performed at week 12; however, different scales/scores were used. Among them, the most commonly used were the O’Leary–Sant Symptom and Problem Index (ICSI/ICPI), the VAS pain assessment and the Pain, Urgency and Frequency (PUF) score.

**Fig. 2:**
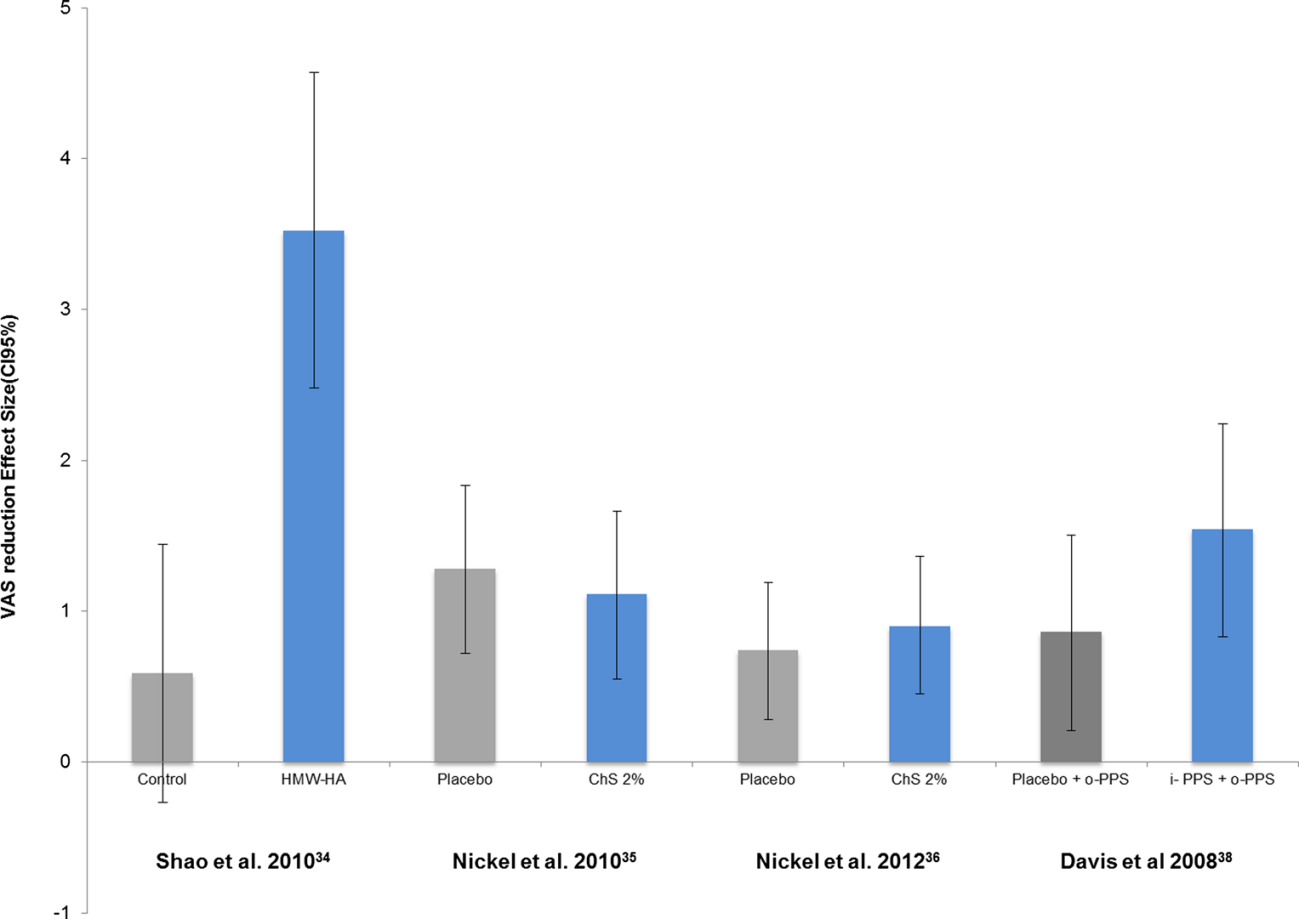
Randomised controlled trials: effect size of average VAS reduction. *95*%*CI* 95 % confidence intervals, *HMW-HA* high molecular weight hyaluronic acid 0.08 %, *ChS 2* % chondroitin sulphate 2 %, *o-PPS* oral dose of pentosan polysulphate, *i*- *PPS* intravesical instillation with pentosan polysulphate

According to their design, studies were classified as controlled (Table 3) and uncontrolled (Table 4) trials. All the studies reviewed reported reductions of VAS pain scores after treatment, including those controlled using placebo/inactive controls (Table 3). The ES of VAS reduction was calculated for each CT and showed significant differences between active and control groups in all but one study, which compared ChS 2 % with a placebo arm [36]. The largest ES in all CT studies was observed for HMW-HA in the study by Shao et al. (Fig. 2) [34], with similar findings in two UCT studies with HMW-HA [16, 40] by a superior “d” for average VAS difference. Closest to this VAS reduction were the results obtained using 300 mg of intravesical PPS [47].

**Table 3:** Effect size of the average reduction on the VAS pain (12 weeks) and the response rates for controlled trials (CT)

Study	Treatment	Total patients	Total instillations at week 12	VAS BT		VAS AT		VAS ↓	RR (%)	VAS effect size			RR effect size		
				Mean	SD	Mean	SD			d	Minimum	Maximum	d	Minimum	Maximum
Shao et al. [34]	Control	11	6	7.1	1	6.6	0.7	0.50	18.2	0.59	−0.27	1.44	0.88	0.01	1.76
	Cystistat®	18	6	7.1	1.1	3.4	1	3.70	93.3	3.52	2.48	4.57	2.62	1.73	3.51
Lai et al. [41]	Cystistat®	29	9	3.28	2.45	2.13	2.67	1.15	69.0	0.45	−0.07	0.97	1.77	1.16	2.38
	Cystistat®	30	12	3.3	2.38	2.0	2.02	1.30	70.0	0.59	0.07	1.11	1.77	1.17	2.37
Nickel et al. [35]	Placebo	30	6	6.2	1.31	4.1	1.98	2.10	23.3	1.28	0.72	1.83	1.01	0.47	1.54
	Uracyst®	29	6	6.5	1.33	4.8	1.74	1.70	41.45	1.11	0.55	1.66	1.4	0.82	1.97
Nickel et al. [36]	Placebo	40	8	6.38	1.83	4.66	2.84	1.72	31.3	0.74	0.28	1.19	1.95	1.42	2.49
	Uracyst®	41	8	6.5	1.81	4.35	2.95	2.15	38	0.9	0.45	1.36	1.81	1.3	2.33
Bade et al. [37]	Placebo	10	24	–	–	–	–	–	20.0	–	–	–	0.93	0.0	1.85
	i-PPS 300 mg^b^	9	24	–	–	–	–	–	40.0	–	–	–	1.46	0.42	2.5
Davis et al. [38]^a^	Placebo + daily o-PPS 400 mg	20	36	4.7	1.3	3.2	2.2	1.50	90.0	0.86	0.21	1.50	2.5	1.67	3.33
	i-PPS 200 mg^c^ + daily o- 400 mg	20	36	4	0.7	2	1.9	2.00	85.7	1.54	0.83	2.24	2.37	1.58	3.15
Sairanen et al. [39]	DMSO	37	6	6.4	2.1	–	–	–	30.0	1.08	0.59	1.56	1.16	0.67	1.65
	BCG	31	6	6.8	2.1	–	–	–	11.0	–	–	–	0.68	0.21	1.14

*VAS* visual analogue pain score, *BT* before treatment, *AT* after treatment, *RR* response rate, *d* Cohen’s d, *i-PPS* intravesical PPS, *o-PPS* oral PPS, *DMOS* dimethyl sulfoxide, *BCG* intravesical Bacillus Calmette–Guérin

^a^Results at 24 weeks

^b^Intravesical instillation with 300 mg (three capsules) of Elmiron® + mixed with 50 ml of 0.9 % sodium chloride

^c^200 mg or two capsules mixed with 30 ml of sterile normal buffered saline

**Table 4:** Effect size results for uncontrolled trials (UCT)

Study	Treatment	Total patients	Number of instillations (12 weeks)	VAS BT		VAS AT		VAS ↓	RR (%)	VAS effect size			RR effect size		
				Mean	SD	Mean	SD			d	Min	Max	d	Min	Max
Morales et al. [15]	0,8 % HMW-HA (Cystistat®)	25	6	6.7	2.45	2.7	3.67	4.00	71.0	1.31	0.70	1.92	2.00	1.32	2.68
Kallestrup et al. [42]		20	6	4.7	2.3	3.3	3.0	1.40	65.0	0.53	−0.10	1.16	1.88	1.13	2.62
Gupta et al.^a^ [43]		20	6	–	–	–	–	–	55.6	–	–	–	1.68	0.96	2.40
Riedl et al. [16]		121	12	8.5	1.7	3.5	2.7	5.00	85.0	2.27	1.95	2.60	2.35	2.02	2.67
Engelhardt et al. [40]		48	10	8.15	1.7	2.71	1.96	5.44	85.0	2.97	2.39	3.55	2.35	1.83	2.87
Steinhoff^b^ [44]	0.2 % CS (Gepan instill®)	13	–	–	–	–	–	–	92.3	–	–	–	2.58	1.83	3.33
Nordling and van Ophoven [45]		165	8	5.2	2.57	3.3	2.57	1.90	76.7	0.74	0.52	0.96	2.13	1.86	2.4
Nickel et al. [46]	2 % CS (Uracyst®/Uropol S®)	53	10	6.9	1.8	4.3	2.3	2.60	60.0	1.27	0.85	1.69	1.77	1.32	2.22
Porru et al. [24]	1,6 % HA+ 2 % CS (Ialuril®)	23	12	5.4	2.8	3.6	2.5	1.80	46.0	0.68	0.08	1.27	1.49	0.84	2.14
Porru et al. [47]		20	10	5.6	2.3	3.2	3.1	2.40	53.48	0.89	0.24	1.54	1.64	0.92	2.36
Bade et al. [48]	i-PPS 300 mg	6	24	7.5	1.38	4.17	2.3	3.33	66.7	1.81	0.47	3.15	1.91	0.55	3.28
Daha et al. [17]		25	22	–	–	–	–	–	16.0	–	–	–	0.82	0.25	1.4

*VAS* visual analogue pain score, *BT* before treatment, *AT* after treatment, *RR* response rate, *d* = Cohen’s d, *i-PPS* intravesical instillation with 300 mg (three capsules) of Elmiron® + mixed with 50 ml of 0.9 % sodium chloride

^a^6 weeks

^b^24 weeks

**Fig. 3:**
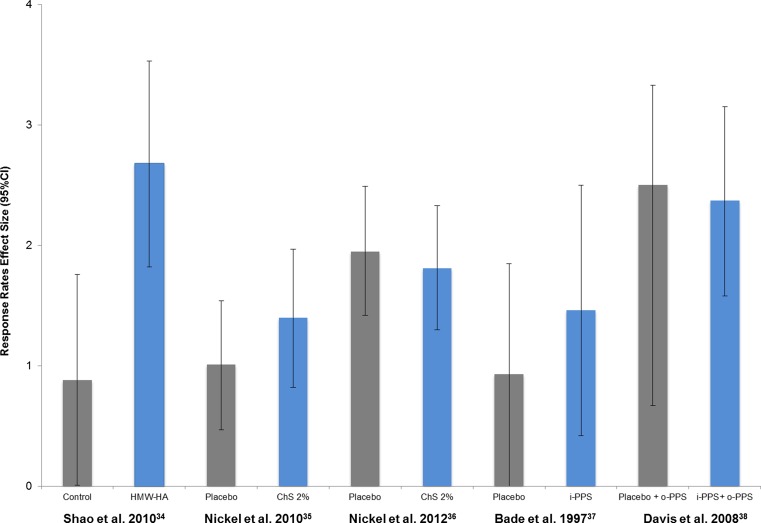
Randomised controlled trials: effect size of the response rates. *95*%*CI* 95 % confidence intervals, *HMW-HA* high molecular weight hyaluronic acid 0.08 %, *ChS 2* % chondroitin sulphate 2 %, *o-PPS* oral dose of pentosan polysulphate, *i*- *PPS* intravesical instillation with pentosan polysulphate

Response rates were compared between intervention arms by ES assessment only in the trial by Shao et al. [34] with HMW-HA (d = 2.68 [IC95%:1.82–3.53] vs d = 0.88 [IC95%:0.01–1.76]) for patients without intravesical therapy (Fig. 3). In CTs, studies with HMW-HA and with ChS 0.2 % reported superior response rates, with both rates (HMW-HA vs ChS 0.2 %) not being statistically different.

**Fig. 4:**
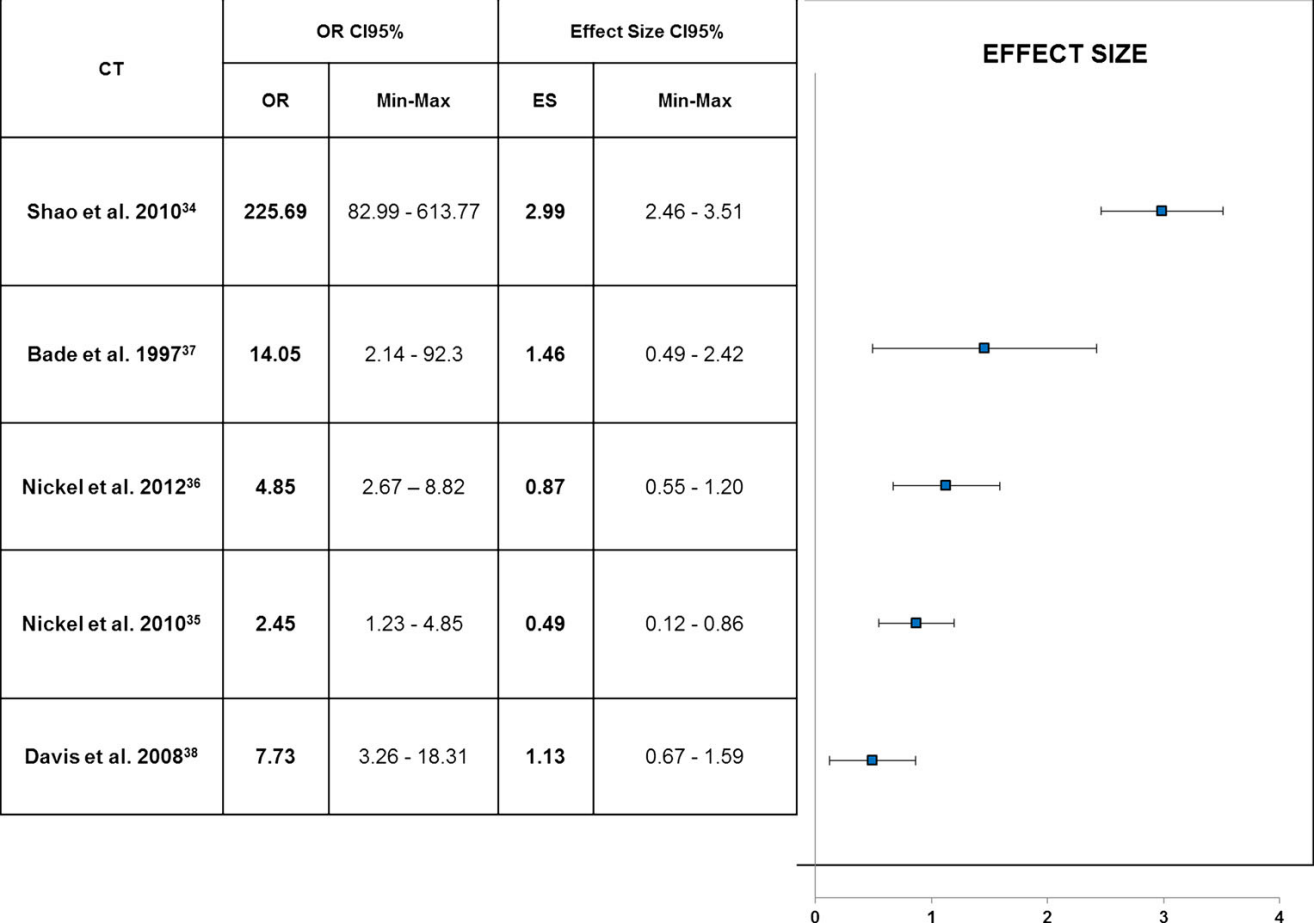
Composite effect size of response rates and VAS reduction: active treatments versus placebo (OR 95%CI). *95*%*CI* 95 % confidence intervals, *VAS* visual analogue scale of pain, *OR* odds ratio

The post-hoc calculation of composite ES based on VAS improvement plus RR revealed distinct differences among studies and products as shown in Fig. 4. OR value was highest for HMW-HA in the Shao et al. study [34], followed by the intravesical application of 300 mg of PPS described by Bade et al. [37].

### Pharmacoeconomic evaluation/cost-effectiveness

The NNT for a response to intravesical therapy ranged between 1.33 (HMW-HA) and 14.81 (ChS 2 %), with a negative value for the PPS combination therapy (Table 5). With this low NNT, HMW-HA was also significantly superior with regard to cost-effectiveness and cost efficacy to all other instillation regimes.

**Table 5:** Pharmacoeconomic evaluation

Product	HMW-HA 0.08 % (Cystistat®)	2 % CS (Uracyst®-Uropol S®)		i-PPS 300 mg (Elmiron®)	i-PPS 200 mg + o-PPS 400 mg (Elmiron®)
Study	Shao et al. [34]	Nickel et al. [35]	Nickel et al. [36]	Bade et al. [37]	Davis et al. [38]
Cost per instillation	1	1	1	0.4	0.6
Number of instillations	6	6	8	24	36
Cost of treatment (CT)	6	6	8	9.6	21.6
%Responders placebo/control	0.182	0.233	0.313	0.200	0.90
%Responders active treatment	0.933	0.4145	0.38	0.40	0.857
Odds ratio (95%IC)	76.5 (6.08; 963.06)	2.33 (0.76; 7.17)	1.35 (0.58; 3.11)	3.19 (0.42; 24.38)	0.67 (0.10; 4.48)
Absolute risk reduction (95%CI)	− 0.75 (−1–0; −0.50)	−0.18 (−2.88; 0.18)	−0.07 (−0.26; 0.12)	−0.20 (−0.59; 0.19)	0.04 (−0.16; 0.24)
NNT (95%CI)	1.33 (1.0; 2.0)	5.51 (2.4; 18.84)	14.81 (3.92; 8.31)	2.67 (0.36; 19.71)	−23.33 (−4.13; 6.40)
Cost-effectiveness (CT/%responders)	6.43	14.49	21.05	21.62	25.20
Cost efficacy (CT ^a^NNTs)	7.98	33.06	118.48	25.63	503.93

*HMW-HA* high molecular weight hyaluronic acid, *CS* chondroitin sulphate, *i-PPS* intravesical pentosan polysulphate, *o-PPS* oral pentosan polysulphate, *NNT* number needed to treat to obtain a response

^a^Response rates correspond to those presented in the articles [29, 30, 31, 33] and/or calculated by total of responders/total patients with per protocol results [32]

## Discussion

The present meta-analysis on intravesical BPS/IC therapy clearly demonstrates the dilemma of the poor scientific evidence currently available for this disease. Owing to the different associations regarding the aetiology of the disease and its rather low prevalence, most published studies cohorts are heterogeneous. The lack of globally accepted instruments for the evaluation of treatment success resulted in the exclusion of some trials from this meta-analytical review because the reported outcomes were not comparable.

Even after a careful selection of 19 studies, we still found a heterogeneous population of 801 patients (mostly women) who were considered to have BPS/IC according to four different diagnostic criteria and typified as “treatment-refractory” (a concept that is not further defined) in 6 studies or as “treatment-naïve” patients in 1 study. Differences in design are also of particular relevance as only 5 studies (26 %) compared the intravesical formulas against placebo or non-active controls, while the remainder corresponded to uncontrolled and observational trials.

Length of treatment and frequency of instillations also differed: weekly instillations were initially performed in 15 studies, biweekly or twice weekly instillations were reported for 2 studies each, while the duration of instillation therapy varied from 6 weeks to 12 months. Similarly, the time period of follow-up for final evaluation varied from 3 months to 5 years.

All the instruments/scales used for outcome evaluation (O’Leary–Sant Score, the PUF and VAS for pain) can potentially measure treatment effects, but they are not readily comparable with each other. There is also no globally accepted definition as to the percentage of symptom regression that is regarded as treatment response. In addition, relatively small differences in VAS scores before and after treatment may be statistically but probably not clinically significant. With the intention to improve the balance between investigational and clinical outcomes, and to extract the maximal information from the selected set of evaluable studies on intravesical BPS/IC therapy, refined statistical techniques such as Cohen’s *d* along with confidence intervals have been used in this meta-analytical review to be able to compare the selected set of evaluable studies.

Interestingly, symptom improvement was observed in all cases, including those from the placebo/non-active treatment arms. By far the largest effect sizes (d > 2) for symptom reduction were found in 3 studies performed with HMW-HA [16, 34, 40].

With respect to response rates, effect size measurements showed similar results for HMW-HA and 0.2 % ChS, and for PPS. If only CTs were included in the analysis, HMW-HA was significantly superior to all other instillates (Table 3). The closest results on efficacy were observed with intravesical PPS [37] and a combination of intravesical plus oral PPS [38]; this last combined strategy reported by Davis et al. [38], however, showed a higher RR in patients who received oral PPS alone (90 vs 85.7 %).

In the pharmacoeconomic approach of CTs, a clear advantage for HMW-HA was observed: the NNT for a treatment response was 1.31 for HMW-HA vs 2.74 for intravesical PPS and 5.51 for ChS 2 %. Cost efficacy (treatment costs * NNT) and cost effectiveness (treatment costs/responders) were higher for HMW-HA. Cost effectiveness was less than half for ChS and only about a third for PPS compared with HMW-HA, and cost efficacy was less than 25 % for ChS compared with HMW-HA. However, these results are based on a small number of studies with final analysis.

The present meta-analytical analysis adds important information to the body of published evidence and is partly contradictory to systematic reviews that have been published in the past. In particular, ES assessment of outcome parameters facilitates the comparison of results with different GAG products for BPS/IC.

Madersbacher et al. [7] searched the literature for all forms of chronic cystitis, including radiation cystitis and also OAB (which is not considered a form of chronic cystitis) and excluded all but 27 publications for further analysis. They concluded that ChS is superior to other intravesical GAG substitutes. However, in their review, 368 patients who were treated with ChS had a diagnosis of OAB, 20 patients a diagnosis of radiation cystitis [27], and only 118 patients were “exclusively” diagnosed with BPS/IC. The authors also state that no significant superiority versus controls was observed in the single controlled study on ChS 2 % that was reviewed [35].

Giannantoni et al. [49] evaluated CTs and UCTs on a multitude of therapies for BPS/IC, including behavioural, dietary, interventional, pharmacological and surgical therapies. In their systematic review not a single study on HA therapy was included. Given the high number of publications on HA in BPS/IC reporting superiority to other intravesical agents, this review is presumably incomplete. The authors conclude that evidence of BPS/IC therapy is limited, and that only the oral drugs cyclosporine A and amitriptyline showed significant ES on the classical BPS/IC symptoms of pain and frequency/urgency.

Fall et al. [13] reviewed the literature to find an evidence base for treatment decisions in BPS/IC. Their conclusions were: level of evidence (LE) 1b/grade of recommendation (GR) A for intravesical PPS, LE 2b/GR B for HA and for ChS, and LE 3/GR C for intravesical heparin.

The review performed by Matsuoka et al. [50] included four treatment modalities for BPS/IC: resiniferatoxin, Bacillus Calmette–Guerin (BCG), oxybutynin and alkalinised lidocaine; as no GAG substitutes were evaluated, their results are not comparable with our analysis.

In conclusion, this meta-analytical review provides evidence of the positive effects of intravesical GAG therapy for BPS/IC and that this treatment may significantly improve patients’ symptoms. Single reports even suggest that complete and permanent remission is possible in a subgroup of patients that has not yet been well defined [40, 42].

If medical and pharmacoeconomic aspects are combined, HMW-HA seems to have some advantage over other instillation agents. Despite these findings, direct comparisons between the different products have not been performed to date in properly designed controlled studies.

The present meta-analysis suffers from the limited number of controlled accessible studies on intravesical therapies for BPS/IC and non-standardised response criteria. Many studies had to be excluded because of non-comparable inclusion criteria, treatment combinations and evaluation instruments. However, it gives a complete summary of all the data currently available and, by assessing the effect size, makes it possible to compare the relevance of the individual studies.

## Abbreviations

BPS/IC: Bladder pain syndrome/interstitial cystitis
ChS: Chondroitin sulphate
CT: Controlled trial
DMSO: Dimethyl sulfoxide
ES: Effect size
ESSIC: International Society for the Study of BPS
FDA: Food and drug administration
GAG: Glycosaminoglycans
HA: Hyaluronic acid
HMW: High molecular weight
LMW: Low molecular weight
MW: Molecular weight
NIDDK: National Institute of Diabetes and Digestive and Kidney Diseases
NNT: Number needed to treat
PPS: Pentosan polysulphate
PST: Potassium sensitivity test
RR: Response rate
UCT: Uncontrolled trial
VAS: Visual analogue scale
